# Cardiovascular complications in vascular connective tissue disorders after COVID-19 infection and vaccination

**DOI:** 10.1371/journal.pone.0315499

**Published:** 2024-12-20

**Authors:** Anthony L. Guerrerio, Allyson Mateja, Gretchen MacCarrick, Jonathan Fintzi, Erica Brittain, Pamela A. Frischmeyer-Guerrerio, Harry C. Dietz

**Affiliations:** 1 Division of Gastroenterology, Hepatology and Nutrition, Department of Pediatrics, Johns Hopkins University School of Medicine, Baltimore, Maryland, United States of America; 2 Clinical Monitoring Research Program Directorate, Frederick National Laboratory for Cancer Research. Frederick, Maryland, United States of America; 3 McKusick-Nathans Institute of Genetic Medicine, Johns Hopkins University School of Medicine, Baltimore, Maryland, United States of America; 4 Biostatistics Research Branch, National Institute of Allergy and Infectious Diseases, National Institutes of Health, Bethesda, Maryland, United States of America; 5 The Laboratory of Allergic Diseases, National Institutes of Allergy and Infectious Diseases, National Institutes of Health, Bethesda, Maryland, United States of America; 6 Howard Hughes Medical Institute, Chevy Chase, Maryland, United States of America; Public Library of Science, UNITED KINGDOM OF GREAT BRITAIN AND NORTHERN IRELAND

## Abstract

**Background:**

COVID-19 infection and vaccination have been reported to confer an elevated risk for cardiovascular events (CVE). We sought to determine whether individuals with an underlying vascular connective tissue disorder including Marfan syndrome (MFS), Loeys-Dietz syndrome (LDS), or vascular Ehlers Danlos syndrome (vEDS) are at increased risk for cardiac events after COVID-19 infection or vaccination.

**Methods:**

325 respondents self-reported data through a cross-sectional, web-based survey available from 22 November 2021, through 15 March 2022 regarding COVID-19 illness and vaccinations, the occurrence of any CVE, and adverse events following vaccination. The data were analyzed using a Cox proportional hazards model with time varying indicators for COVID-19 illness/vaccination in the preceding 30 days.

**Results:**

COVID-19 illness was significantly associated with an increased rate of a new abnormal heart rhythm 30 days following infection. No other CVEs were reported in the 90 days after COVID-19 illness. We did not find evidence of an increased rate of any CVE in the 30 days following any COVID-19 vaccination dose.

**Conclusion:**

In respondents with MFS, LDS, or vEDS, we uncovered no evidence of an increase in CVEs in the 30 days following COVID-19 illness, with the possible exception of dysrhythmia. In light of the absence of a substantial increase in self-reported CVEs in the 30 days following COVID-19 vaccination, these data are in keeping with the recommendation from the Marfan Foundation Professional Advisory Board that all eligible persons be vaccinated for COVID-19.

## Introduction

The heritable vascular connective tissue disorders Loeys-Dietz syndrome (LDS), Marfan syndrome (MFS), and vascular Ehlers-Danlos syndrome (vEDS) share a predisposition for arterial aneurysm, dissection, and/or rupture. Both structural and cellular signaling abnormalities in the arterial wall play a role in increasing the susceptibility for these vascular events [1–3]. While inflammation is a well-known factor in the development and susceptibility to rupture of non-genetic aortic aneurysms [4], emerging evidence indicates that inflammation may also play a role in the pathogenesis and progression of aortic aneurysms in the setting of genetic syndromes [5]. Cardiovascular manifestations have been recognized as a complication of SARS-CoV-2 infection and are the most-common non-pulmonary manifestation of infection [6, 7]. While the mechanism remains unclear, numerous studies have proposed an association between SARS-CoV-2 infection and new or worsening vascular disease including aneurysm formation, progression, dissection and/or rupture—prominently including involvement of the aorta and cerebral vasculature [8–10]. Furthermore, other forms of cardiovascular morbidity variably associated with COVID-19 illness or preventative immunization such as arrhythmia or myocardial or vasomotor dysfunction are more common in individuals with vascular connective tissue disorders compared to the general population [11–13].

SARS-CoV-2 directly infects vascular endothelial cells through first attaching to the angiotensin-converting enzyme 2 (ACE2) receptor [14] leading to cellular damage and apoptosis of the endothelium [15, 16]. The resulting endothelial dysfunction promotes ischemia, inflammation and a procoagulant state [17]. While a significant body of research has investigated the pro-thrombotic effects of SARS-CoV-2 infection, there are also significant inflammatory effects as well as overexpression of many factors including vascular endothelial growth factor (VEGF), VEGF receptor 1 (Flt-1), intracellular adhesion molecule 1 (ICAM-1), matrix metalloproteinase 2 (MMP-2), platelet-derived growth factor (PDGF), TNFα, and TGFβ [18, 19]. Many of these molecules have been shown to play a role in aneurysm formation and progression in general and in heritable vascular connective tissue disorders specifically [20–26], suggesting a mechanism for how infection might lead to aneurysm complications in these disorders.

Similar to SARS-CoV-2 infection, vaccines for COVID-19, while they provide protection against severe disease and death, have also been linked to an elevated risk of cardiovascular and cerebrovascular complications [27–30]. On this basis, we posited that the local or systemic inflammatory response that can be seen with SARS-CoV-2 infection or immunization might have more frequent or severe cardiovascular sequelae in individuals with an underlying genetic predisposition for impaired cardiovascular homeostasis, as seen in MFS, LDS and vEDS.

In addition to the documented risk of cardiovascular complications, COVID-19 mRNA vaccinations initially appeared to have a low but elevated risk of inducing an anaphylactic reaction compared to other vaccines, with COVID-19 mRNA vaccines having 3.29–5.58 anaphylactic reactions/million doses [31, 32] compared to 1.3 anaphylactic reactions/million doses for other vaccines [33]. As patients with LDS have been shown to be at increased risk for allergic diseases [34], we also sought to determine if individuals with LDS were likely to have an allergic reaction to COVID-19 vaccines.

Previous work has examined the susceptibility to complications in hypermobile Ehlers-Danlos Syndrome (hEDS) to COVID-19 vaccination and illness [35]. However, there is no known underlying susceptibility in hEDS to vascular wall pathology as there is in MFS, LDS and vEDS, and no susceptibility to the development of allergies as in LDS(34); therefore, we conducted a separate analysis of this population.

## Materials and methods

### Study design and setting

This was a cross-sectional web-based survey. The survey questions are included as Appendix 1 (Data Dictionary). Respondents were asked to provide information regarding the following: 1) Diagnosis and demographics; 2) date of a COVID-19 positive test/diagnosis and disease severity; 3) dates/type of COVID-19 vaccination received; 4) symptoms and timing/treatment of symptoms after vaccination; 5) occurrence of any cardiovascular event (CVE). Respondents were asked to report CVEs that occurred between January 1, 2019, and the date of survey completion. CVEs were chosen as those reported in the literature as possibly related to COVID illness and vaccination as well as complications seen in connective tissue disorders as defined by a connective tissue disorders genetics expert and are listed in Table 1. Respondents were asked about seventeen post vaccination symptoms. For analysis, these were categorized into two classes: expected adverse events (EAE) and symptoms that suggested a possible allergic reaction (SSPAR—see Table 1). These symptoms were chosen for several reasons. First expected adverse events (EAE) were those symptoms asked during the COVID vaccine trials [36]. Given the high rate of allergic reactions reported to COVID vaccination, we also chose to ask subjects about symptoms that might be suggestive of allergic events. These symptoms were chosen to be consistent with the Second National Institute of Allergy and Infectious Disease/Food Allergy and Anaphylaxis Network (NIAID/FAAN) symposium definitions [37]. The questionnaire was reviewed by experts in connective tissue disorders, allergy, and REDCap questionnaire design and was implemented with pre-study testing.

**Table 1:** Categories of events assessed by the questionnaire. Cardiovascular events and symptoms after vaccination. Symptoms were categorized into Expected Adverse Events (EAE) and those that might be suggestive of an allergic reaction.

Categories of Cardiovascular Events (CVEs)	Symptoms classified as expected adverse events (EAE)	Symptoms that suggest a possible allergic reaction (SSPAR).
1. new aneurysm 2. new dissection 3. vascular rupture 4. heart inflammation (myocarditis) requiring hospitalization 5. new abnormal heart rhythm 6. cardiovascular surgery 7. death due to a proven cardiovascular event 8. sudden unexplained death.	1. Nausea, vomiting, diarrhea, abdominal cramps 2. Redness/swelling at injection site 3. Fever 4. Chills 5. Fatigue 6. Body and/or muscle aches (myalgia) 7. Joint pain (arthralgia) 8. Headache	1. Itching, hives, rash 2. Swelling of face/lips/eyes/throat/tongue 3. Feeling of throat itching/scratchiness/closing, lump in throat 4. New hoarseness of voice 5. Runny nose, itchy eyes 6. Coughing spells, wheezing, shortness of breath/difficulty breathing 7. Palpitations (heart racing), dizziness 8. Passing out, loss of consciousness 9. Low blood pressure, hypotension.

### Study and source population

A web-based survey was advertised through the Marfan Foundation website (serving the Marfan Foundation, Loeys-Dietz Syndrome Foundation, and the vEDS Movement) and made available from November 22, 2021, through March 15, 2022. Responses were not restricted based on ISP location, although participants were asked if they were located in the United States and questions indicated the study would be asking about vaccines available in the United States. Respondents could enter data for themselves and/or family members. Diagnoses were self-reported. Parents/guardians were instructed to fill out the survey for participants less than 18 years of age. During the survey period, COVID-19 vaccinations for younger children became available, and parents/guardians were asked to wait at least eight weeks after the second vaccination to fill out the survey for children less than 12 years of age.

### Data collection tool and procedure

Advertised web address directed respondents to a REDCap [38, 39] based data collection tool into which respondents entered their own data.

### Data quality control

Entries with no date entered for any event were removed. Participants were asked if they had ever entered data for the survey before. Those answering yes were further manually inspected and in the two cases they could be resolved due to repeated email/phone number and/or multiple exact dates of events. Respondents were asked to provide as much information regarding the date of events as they could recollect. When provided, the exact date was used for analysis. If only the month and year were provided, the date was imputed as the 15th of the month. If only the year was provided, the date was considered missing. If subjects were unable to provide the exact date but provided information that two events occurred in the same month, they were given the opportunity to temporally order the events. Data were excluded from analysis if COVID-19 illness was reported to occur prior to March 1, 2020; an event was specified to occur after the survey completion date; a vaccination was reported to occur prior to December 1, 2020; or two COVID-19 vaccinations were reported on the same day.

### Ethical consideration

All procedures performed in studies involving human participants were in accordance with the ethical standards of the institutional and/or national research committee and with the 1964 Helsinki declaration and its later amendments or comparable ethical standards. This study was approved by the Johns Hopkins institutional review board as exempt research under the DHHS regulations. As this was exempt research no specific consent was required, however the front matter to the survey contained the statement: “Answering the survey questions implies consent for participation in this study.”

### Statistical analysis

To determine if COVID-19 illness or vaccination was associated with an increase in CVEs, we used a Cox proportional hazards model with time varying indicators for COVID-19 illness or vaccination in the previous 30 days. The dependent variable is a CVE. The independent variable is COVID-19 illness or vaccination in the previous 30 days. Evidence of an increase in CVEs in the 30 days following COVID-19 or vaccination was assessed via a Wald test of the estimated effect of each time-varying covariate. The start of the modeling period was January 1, 2019, and participants who did not experience a CVE were censored on the date of survey completion; all participants were censored by March 15, 2022. Hence, each respondent contributed either a CVE date or a censoring time to the data. The Cox model was fit separately for all CVEs, new abnormal heart rhythm, and the group of any CVE other than new abnormal heart rhythm (new aneurysm, new dissection, vascular rupture, myocarditis requiring hospitalization, cardiovascular surgery, or death). Analysis was performed using STATA [40] and R [41].

## Results

### Demographics

There were 336 respondents. After removing entries with no dated events and merging two respondents who provided duplicate submissions, 325 respondents provided data of sufficient quality for inclusion in analysis, including 118 with LDS (75 female), 176 with MFS (119 female), and 31 with vEDS (26 female) (Table 2). Subjects with other genetic aortic aneurysm syndromes did not respond in significant numbers and were excluded. The mean age (range) for LDS, MFS, and vEDS respondents was 36.8 (0.01–76.2), 37.1 (0.3–71.6) and 40.1 (9.9–72.1) years, respectively.

**Table 2:** Summary of respondents’ demographics and COVID-19 illness/vaccination information. This table reflects the counts of subjects that indicated in the survey that they had COVID-19 illness or received vaccine doses, regardless of whether or not a date was provided.

	LDS		MFS		vEDS	
Total	118		176		31	
Female	75 (63.6%)		119 (67.6%)		26 (83.9%)	
Mean±SD [min, max] age (y)	36.8 ± 19.4 [0.01, 76.2]		37.1 ± 18.1 [0.3, 71.6]		40.1 ± 18.5 [9.9, 72.1]	
COVID-19 illness*	Yes	No	Yes	No	Yes	No
	44 (37.3%)	74 (62.7%)	45 (25.6%)	131 (74.4%)	6 (19.4%)	25 (80.6%)
COVID-19 No Vaccine doses	16 (13.6%)	6 (5.1%)	12 (6.8%)	4 (2.3%)	1 (3.2%)	2 (6.5%)
COVID-19 Vaccine dose 1	27 (22.9%)	62 (52.5%)	31 (17.6%)	111 (63.1%)	5 (16.1%)	18 (58.1%)
COVID-19 Vaccine dose 2	24 (20.3%)	57 (48.3%)	28 (15.9%)	104 (59.1%)	4 (12.9%)	16 (51.6%)
COVID-19 Vaccine dose 3	10 (8.5%)	37 (31.4%)	14 (8.0%)	66 (37.5%)	1 (3.2%)	9 (29.0%)
Total Vaccine Doses	217		354		53	

* Sum of COVID-19 No Vaccine Doses and COVID-19 Vaccine Dose 1 may not sum to the number of those with COVID-19 illness due to missing data.

### COVID-19 illness

Ninety-five of the 325 participants reported a COVID-19 illness, including 37.3% (44/118) of those with LDS, 25.6% (45/176) with MFS, and19.4% (6/31) with vEDS. Of those 95, 93 were treated at home and two (both with LDS) were admitted to the hospital. Neither required intensive care unit admission.

### COVID-19 vaccination

#### Number and dates of vaccinations

At least one dose of a COVID-19 vaccine was received by 75.4% (89/118) respondents with LDS, 80.7% (142/176) with MFS, and 74.2% (23/31) with vEDS. 32% of the doses were manufactured by Moderna, 64% by Pfizer, 2% by Johnson and Johnson and 2% by an unknown/unspecified source.

Vaccination dose needed an assignable date to use that event for further analysis. A total of 254 subjects received vaccination dose 1 (191 knowing the exact date and 25 set to missing), 233 received dose 2 (174 knowing the exact date and 27 set to missing), and 137 received dose 3 (107 knowing the exact date and 11 set to missing).

#### Expected adverse events (EAEs) after vaccination

**Fig 1:**
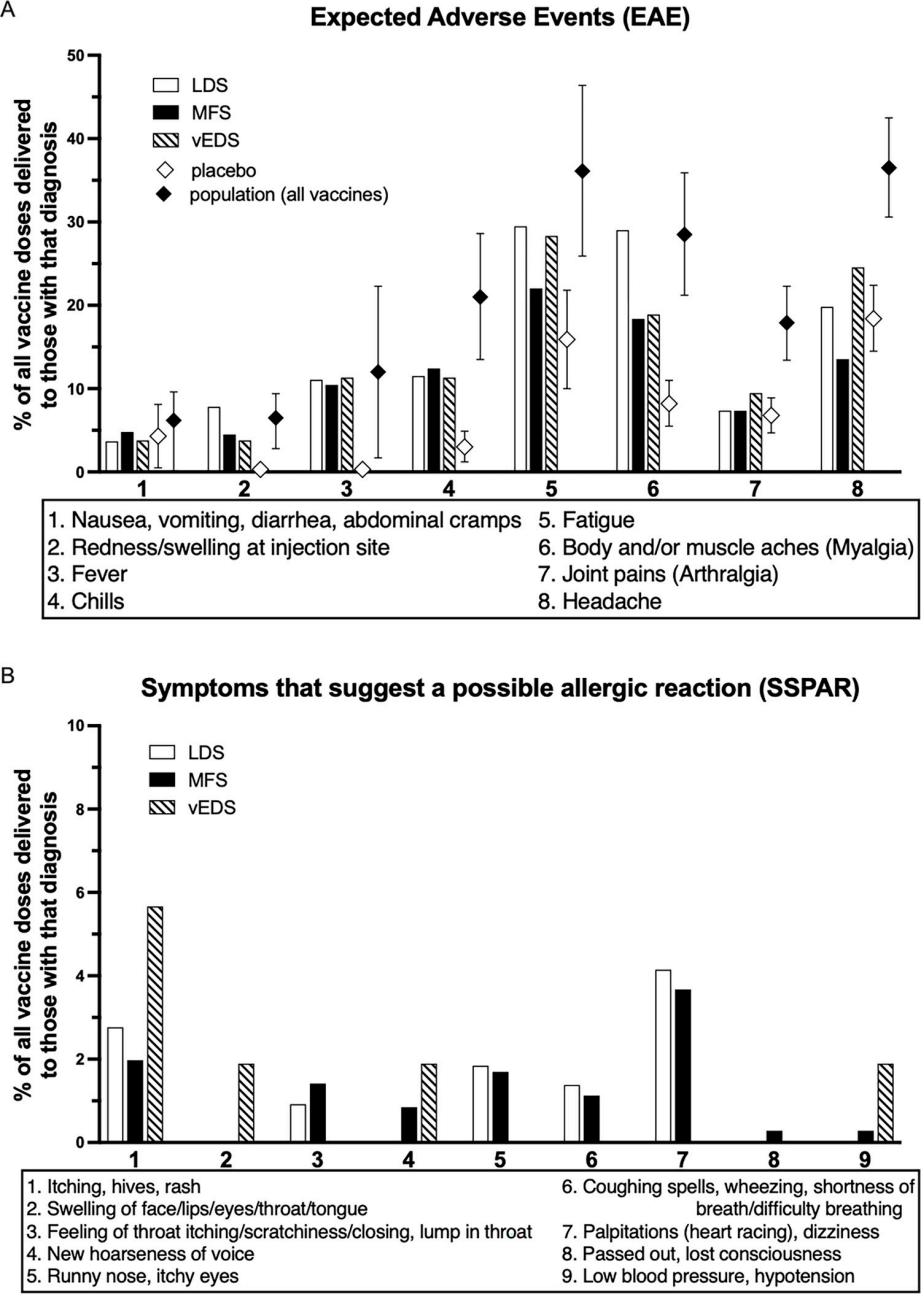
**(A)** Percent of vaccine doses resulting in adverse events in respondents compared to the general population as reported in reference 8. **(B)** Percent of vaccine doses resulting in symptoms suggestive of a possible allergic reaction.

Of the respondents who received at least one dose of a COVID-19 vaccine, 47.4% (154/254) reported at least one vaccination with an EAE, including 59.6% (53/89) of those with LDS, 61.3% (87/142) of those with MFS, and 60.9% (14/23) of those with vEDS. Taking into account multiple vaccinations per person, 38.2% (83/217) of vaccinations in LDS were followed by an EAE, 47.5% (168/354) in MFS, and 34.0% (18/53) in vEDS. The most common EAE reported was fatigue which occurred at a similar percentage across the different types of genetic syndromes studied. Respondents did not have a higher-than-expected number of any EAE compared to the general population [7] (Fig 1A).

#### Symptoms that suggest a possible allergic reaction (SSPAR) after vaccination

When considering SSPAR, of the patients with a specific syndrome, 15.7% (14/89) of those with LDS, 15.5% (22/142) with MFS, and 13.0% (3/23) with vEDS reported at least one vaccination that was followed by a SSPAR. Considering separate vaccination events in each syndrome: 8.3% (18/217) of vaccinations in LDS respondents resulted in SSPAR, 8.5% (30/354) in MFS, and 7.5% (4/53) in vEDS (Fig 1B).

Given this study’s survey nature, no independent verification of allergic reaction or laboratory measurements (e.g., tryptase levels) were obtained. Thus, other variables were considered to determine if a described reaction was likely to represent an allergic reaction. IgE mediated reactions secondary to an injection typically occur in seconds to minutes, but in rare occasions can occur after 4–6 hours. Further, IgE mediated reactions are stereotypical and expected to occur with similar symptomatology with repeated exposure. Of the 18 SSPARs after vaccinations in respondents with LDS, two occurred within an hour of administration and six within six hours. Five of these reactions were not treated and three were treated with over-the-counter pain relievers or home remedies. No respondents with LDS had recurrence of the same SSPAR following multiple vaccinations. Among MFS respondents, eight SSPARs occurred within one hour and 11 within six hours of vaccination. Two patients with MFS developed rash and itching (treated with an antihistamine) within less than 1 hour of receiving the vaccine that recurred following a second vaccination. One MFS participant had two reactions with similar symptomatology at less than six hours (subjective tachycardia treated with an over-the-counter pain reliever at home). No other MFS subjects had the same SSPAR at multiple vaccinations. In the vEDS subgroup, one reaction developed within one hour and one within six hours, but neither participant experienced the same SSPAR after subsequent vaccinations.

#### Cardiovascular events (CVEs)

We next looked at CVEs in the survey group. There was a total of 89 CVEs reported in 71 participants from January 1, 2019, through March 15, 2022 (See Table 3). To use the reported CVEs for further analysis, respondents needed to supply an exact date or a partial date that could be resolved as described in the methods. 51 respondents supplied an exact date, 9 supplied at least a partial date that could be resolved, and 11 respondents did not supply any date for their CVE. This resulted in 60 respondents who had a CVE with a date assigned. Of these 60 CVEs, 14 occurred prior to the onset of the pandemic (before March 1, 2020), and 46 had a CVE that occurred after. The most common CVE (23 of the 89 CVEs) was new abnormal heart rhythms.

**Table 3:** Cardiovascular events reported between January 1, 2019 and March 15, 2022. A total of 89 CVEs were reported. However only 60 respondents reported a CVE’s with a date. Number of CVEs that had a date usable for analysis are listed in parentheses.

	New aneurysm	New dissection	Vascular rupture	Heart inflammation (myocarditis) requiring hospitalization	New abnormal heart rhythm	Cardiovascular surgery	Death due to a proven cardiovascular event	Sudden, unexplained death
LDS	4 (3)	9 (7)	2 (2)	1 (1)	15 (12)	9 (6)	1 (1)	2 (1)
MFS	11 (11)	6 (6)	1 (0)	0 (0)	9 (9)	17 (11)	0 (0)	0 (0)
vEDS	0 (0)	0 (0)	0 (0)	0 (0)	2 (2)	0 (0)	0 (0)	0 (0)

#### Cox proportional hazards model analysis

**Fig 2:**
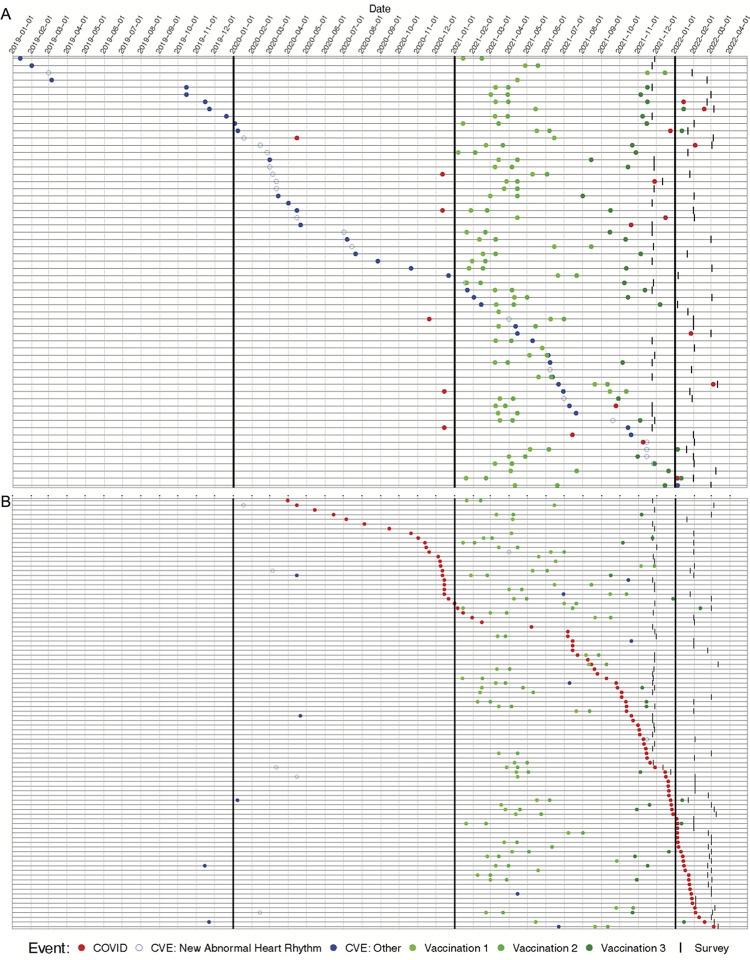
Respondents with **(A)** a reported CVE (ordered by time of CVE), **(B)** a reported positive COVID-19 test/diagnosis (ordered by time of COVID-19 positive test/diagnosis). Horizontal lines represent individual subjects, vertical lines separate months, and symbols denote when COVID (red), CVE (open blue: new abnormal heart rhythm; closed blue: other), vaccination (green; light to dark represents doses 1–3), and survey completion (tick mark) occurred. For example, the first row denotes a survey respondent with a CVE (other than new abnormal heart rhythm) in January 2019, two vaccines in January and February 2021, and survey completed in November 2021. This individual appears in the top line because they had the earliest CVE among the respondents.

**Fig 3:**
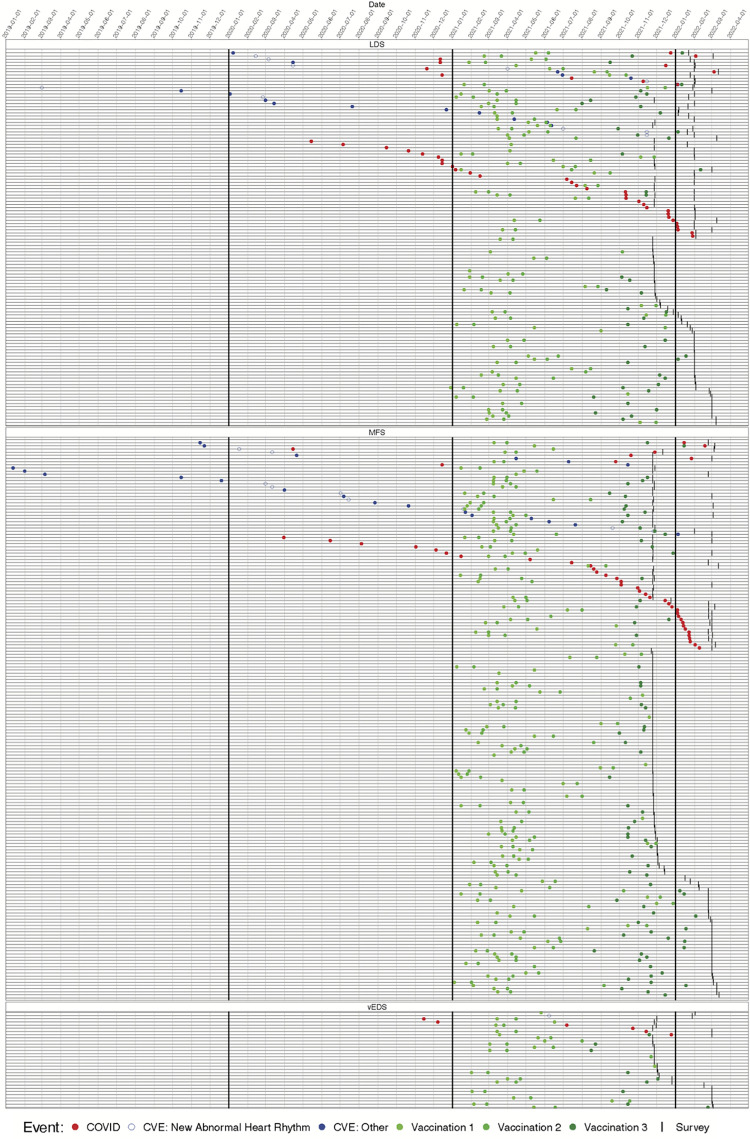
Similar to Fig 2 but showing all respondents grouped by underlying genetic disorder. Subjects are ordered in the following way: subjects with CVE and COVID, ordered by time of CVE; subjects with CVE only, ordered by time of CVE; subjects with COVID only, ordered by time of COVID, subjects without any CVE or COVID, with at least one dose of vaccine, ordered by time of survey completion). Horizontal lines represent individual subjects, vertical lines separate months, and symbols denote when COVID (red), CVE (open blue: new abnormal heart rhythm; closed blue: other), vaccination (green; light to dark represents doses), and survey completion (tick mark) occurred.

Occurrences of CVE, COVID-19 diagnosis, and vaccination are shown in Figs 2, 3 and Tables 4–6. 90% (54/60) of the subjects with a CVE that had a date assigned also had a vaccination with a date assigned. Report of COVID-19 illness was associated with a report of a new abnormal heart rhythm in the following 30 days compared to other time periods (p = 0.005; Table 7 Model 3). Although statistically significant, this result is driven by only two participants who reported a new abnormal heart rhythm within the 30-day period after having COVID-19. Of the 42 respondents with 90 days or less of follow-up after their COVID-19 illness, none reported CVEs other than new abnormal heart rhythms; the additional 50 respondents with less than 90 days follow-up after their illness also reported no such cases during their abbreviated follow-up. Hence, it was not possible to estimate an association between COVID-19 illness and CVEs other than new abnormal heart rhythms.

**Table 4:** Number of patients with CVEs (with identifiable dates) categorized by COVID illness and number of vaccine doses.

	New abnormal heart rhythm			CVE other than new abnormal heart rhythm			No CVE reported			Any CVE with a missing date			Total
COVID illness*	yes	no	missing	yes	no	missing	yes	no	missing	yes	no	missing	
No Vaccine Doses	1	1	0	4	1	0	28	47	2	2	4	0	90
1 vaccine dose*	2	2	0	0	1	0	7	15	1	0	0	0	28
2 vaccine doses*	3	3	0	4	9	0	22	46	0	1	0	0	88
3 vaccine doses*	2	9	0	3	15	0	13	73	0	0	4	0	119
Total	8	15	0	11	26	0	70	181	3	3	8	0	
Total	23			37			254			11			325

* For this table, a COVID-19 illness or vaccine dose is only counted when a date is assigned for that dose. For example, for the 3 vaccine doses, subjects are only included in that row if they had 3 vaccine doses, all of which have a date assigned (either known or imputed to the 15th of the month, but no doses are unknown). Therefore, counts may not match Table 2.

**Table 5:** Summary of the timing of new abnormal heart rhythms relative to COVID-19 illness and vaccination.

	Number of new abnormal heart rhythms occurring in the listed time period (Number of respondents still being followed at beginning of time period)				
Time Period	Illness	Vaccination Dose 1	Vaccination Dose 2	Vaccination Dose 3	Any Vaccination
**> = 120 days after**	**1 (36)**	**5 (212)**	**5 (181)**	**0 (24)**	**5**
**90-<120 days after**	**0 (42)**	**1 (215)**	**0 (192)**	**0 (47)**	**1**
**60-<90 days after**	**0 (51)**	**0 (220)**	**1 (195)**	**0 (67)**	**1**
**30-<60 days after**	**0 (74)**	**0 (223)**	**0 (201)**	**0 (95)**	**0**
**< 30 days after**	**2 (92)**	**2 (229)**	**0 (206)**	**1 (126)**	**3**
< 30 days before	0 (92)	1 (229)	1 (206)	2 (126)	3
30-<60 days before	0 (92)	0 (229)	0 (206)	2 (126)	2
60-<90 days before	1 (92)	1 (229)	1 (206)	1 (126)	2
90-<120 days before	0 (92)	0 (229)	0 (206)	0 (126)	0
> = 120 days before	4 (92)	11 (229)	9 (206)	5 (126)	12

**Table 6:** Summary of the timing of CVEs other than new abnormal heart rhythm relative to COVID-19 illness and vaccination.

	Number of CVEs other than new abnormal heart rhythms occurring in the listed time period (Number of respondents still being followed at beginning of time period)				
Time Period	Illness	Vaccination Dose 1	Vaccination Dose 2	Vaccination Dose 3	Any Vaccination
**> = 120 days after**	**2 (36)**	**4 (212)**	**2 (181)**	**0 (24)**	**4**
**90-<120 days after**	**1 (42)**	**0 (215)**	**2 (192)**	**0 (47)**	**2**
**60-<90 days after**	**0 (51)**	**2 (220)**	**1 (195)**	**0 (67)**	**2**
**30-<60 days after**	**0 (74)**	**1 (223)**	**1 (201)**	**0 (95)**	**2**
**< 30 days after**	**0 (92)**	**2 (229)**	**2 (206)**	**2 (126)**	**5**
< 30 days before	0 (92)	1 (229)	0 (206)	0 (126)	1
30-<60 days before	0 (92)	2 (229)	2 (206)	0 (126)	4
60-<90 days before	1 (92)	2 (229)	3 (206)	0 (126)	4
90-<120 days before	0 (92)	1 (229)	2 (206)	1 (126)	3
> = 120 days before	7 (92)	17 (229)	15 (206)	16 (126)	21

Bolding indicates time periods of interest in analysis: CVEs that occurred after either COVID-19 illness or vaccination. The number in parentheses under “Illness” refers to the number of subjects reporting COVID-19 illness still being followed at the start of that time period; note this number is not reduced by those who reported a CVE earlier. The number in parentheses under the vaccination dose columns are analogous. For example, there were no reported cases of new abnormal heart rhythm in the 30–60 days following COVID-19 illness, and 74 respondents were still being followed 30 days post COVID-19 illness. In other words, of the 92 total respondents reporting COVID-19 illness, for whom we knew a date of illness, 18 respondents completed the survey within 30 days of reporting COVID-19 illness. Vaccination dose columns are not mutually exclusive. For example, 2 respondents reported a CVE other than new abnormal heart rhythm within 30 days following their first vaccine dose (all respondents reporting vaccine dose 1, 229, were still being followed at the start of this time period). For one of those subjects, their CVE also occurred in the 30 days following vaccine dose 2, and for the other subject, their CVE was 30–60 days following vaccine dose 2. The rows of the “Any Vaccination” column are not mutually exclusive. For example, if a respondent reported a CVE 20 days post vaccine dose 1, 50 days post vaccine dose 2, and 150 days post vaccine dose 3, they would appear in the rows “< 30 days”, “30-<60 days”, and “> = 120 days”. If a subject reported a CVE > 120 days post all 3 doses, they would only be counted once in the “> = 120 days” row.

**Table 7:** Cox proportional hazards model results for COVID-19 illness or vaccination and CVEs. There were no cases of CVEs other than new abnormal heart rhythm in the 90 days following illness, therefore it was not possible to estimate an association between COVID-19 illness and CVEs other than new abnormal heart rhythms.

	Outcome	Risk Factor	HR	Lower 95% CI Bound	Upper 95% CI Bound	p value
Model 1	Time to any CVE	Any vaccination dose in the past 30 days	1.48	0.55	4	0.434
Model 2	Time to CVE other than new abnormal heart rhythm	Any vaccination dose in the past 30 days	2.46	0.77	7.87	0.13
Model 3*	Time to new abnormal heart rhythm	Any vaccination dose in the past 30 days	0.66	0.08	5.31	0.699
		COVID-19 illness in the past 30 days	10.97	2.08	57.73	0.005

* These variables run as a single model

There was not a statistically-significantly increased rate of any CVE within one month after vaccination compared to other time periods. This was true whether CVEs were considered as a whole (Table 7 Model 1), as new abnormal heart rhythm considered alone (Table 7 Model 3), or as all CVEs other than new abnormal heart rhythm (Table 7 Model 2). The cohort of respondents who were vaccinated at some point did report CVEs at a higher rate than unvaccinated; however, this appears to be due, in part, to the correlation of greater age with vaccination. Vaccinated subjects had even greater excess CVE rates relative to unvaccinated subjects before vaccines were available than after (Table 8). Sensitivity analyses controlling for age did not appreciably change the estimated association between vaccination and CVEs (Tables 9 and 10).

**Table 8:** Proportion of respondents with designated subset of CVEs in the given time period. Pre-vaccine is defined as on or before 01/01/2021, and post-vaccine as after 01/01/2021. In this table the proportion of those who received multiple vaccines with CVEs is just as great (if not greater) in the pre-vaccine era than the post-vaccine era, supporting that the increase in CVEs in the vaccinated cohort is due to an inherently higher risk of CVE in participants who received vaccines (potentially due to increased age), as opposed to being caused by the vaccines.

	New abnormal heart rhythm		CVE other than new abnormal heart rhythm		Any CVE		Median Age (years)	Proportion ≤ 12 yo as of 1/1/2021	Total N
	Pre-Vaccine availability	Post-Vaccine availability	Pre-Vaccine availability	Post-Vaccine availability	Pre-Vaccine availability	Post-Vaccine availability			
Any Vaccine	0.047	0.043	0.077	0.06	0.123	0.102	38.7	0.098	235
No Vaccine	0.0	0.022	0.022	0.033	0.022	0.056	30.5	0.222	90

**Table 9:** Sensitivity analysis based on age. Cox proportional hazards model results for COVID-19 illness or vaccination and CVEs with binary age (greater than 12 years old on 1/1/2021) added as a covariate.

	Outcome	Risk Factor	HR	Lower 95% CI Bound	Upper 95% CI Bound	p value
Model 1	Time to any CVE	Any vaccination dose in the past 30 days	1.403	0.518	3.801	0.506
Model 2*	Time to CVE other than new abnormal heart rhythm	Any vaccination dose in the past 30 days	2.269	0.702	7.334	0.171
Model 3**	Time to new abnormal heart rhythm	Any vaccination dose in the past 30 days	0.628	0.078	5.052	0.662
		COVID-19 illness in the past 30 days	11.672	2.189	62.245	0.004

* There were no “CVEs other than new abnormal heart rhythm” in subjects ≤ 12 years old on 1/1/2021 therefore binary age could not be included as a covariate. Instead for the analysis in this row, we restrict to individuals over age 12 years old on 1/1/2021 to again check that the estimates are relatively unchanged from those in Table 8

** These variables run as a single model

**Table 10:** Cox proportional hazards model results for age greater than 12 years old on 1/1/2021.

	HR	Lower 95% CI Bound	Upper 95% CI Bound	p value
Age > 12 yo and any CVE*	9.961	1.38	71.908	0.023
Age > 12 yo and new abnormal heart rhythm**	4.348	0.584	32.381	0.151

* This result derived from the same model presented in row 1 of Table 9

** This result derived from the same model as presented in row 3 of Table 9

## Discussion

COVID-19 illness and vaccination has been associated with an increase in adverse cardiovascular outcomes, prompting concern that patients with underlying cardiovascular disease due to genetic causes may be at higher risk for these sequelae. Here, we evaluated the self-reported experiences with COVID-19 illness and vaccination in individuals with LDS, MFS, and vEDS and did not detect a signal that COVID-19 vaccination was more likely to lead to complications in the 30 days following vaccination in this population. Following COVID-19 illness, we did find an association of a new abnormal heart rhythm in the 30 days following reported COVID-19 illness, we we did not find an increased risk of any other CVEs. Whether the dysrhythmias were short-lived and situational or indicative of sustained pathology could not be ascertained from the survey nature of the study. Additionally, the association was based on relatively few events. While dysrhythmias are reported in patients hospitalized with COVID-19 [42], a definitive proarrhythmic effect of COVID-19 infection, outside of one associated with an acute illness, has not been conclusively shown [43, 44]. In those with long term symptoms after a COVID-19 infection, approximately 10% to two thirds report tachycardia/palpitations [45–48]. In our study, there was no medical confirmation that the reported new abnormal heart rhythm was not simply a tachycardia (and if it was actual or just perceived) as a response to illness or a fever. There was the additional limitation that a new abnormal heart rhythm is more likely to be detected once someone is already receiving medical attention due to COVID-19, increasing the probability of finding an association as was seen in this study. Further, while our association was statistically significant, there were only two participants reporting a new abnormal heart rhythm within the 30-day period after having a COVID-19 illness. These limitations highlight a need for confirmatory evidence of this link.

The roll out of the COVID-19 vaccines was quickly complicated by high rates of reported anaphylactic reactions. Given the underlying predisposition of those with LDS to allergic disease, which has not been reported for MFS or vEDS, we hypothesized that a vaccine with a high allergenic potential may preclude its use in the LDS population. However, we found that respondents with all three connective tissue disorders reported similar rates of SSPAR, most of which were mild and self-limited. Based on the timing of symptoms following vaccination and their lack of recurrence in most cases following subsequent vaccination, most of the reported reactions across participants were unlikely to represent true allergic reactions. More recent studies have shown the rate of anaphylaxis to the COVID-19 mRNA vaccine to be more in line with traditional vaccines [49, 50]. Additional studies have suggested that some of the initial reactions described as anaphylactic may have been immunization stress–related responses [51, 52]. Reassuringly, our data therefore do not suggest that patients with vascular CTD are at greater risk for allergic reactions to the vaccine, including those with LDS.

While the study design did allow data collection on a much larger number of individuals than could be obtained by chart review at a single institution, this study is limited by the exclusive use of self-reported survey data, attendant recall bias, and ascertainment bias. Despite our original concerns and hypotheses, the number of actual CVEs was small. With our study design, we could reliably detect only a large increase in the rate of CVEs following vaccination, and we may have failed to find associations due to limited events, tempering our conclusions. Given that nearly all (98%) of respondents with a COVID-19 infection were treated at home, our study is not inconsistent with a study of veterans in which there was no increased risk of death beyond 6 months after infection among nonhospitalized COVID-19 infected patients [53]. The risk of repeated infection was not fully appreciated at the time of the survey and was not assessed. Since the completion of this study, the cumulative risks of repeat infection have been shown to contribute to an additional risk of death, increasing with the number of infections [54]. This study was not designed to evaluate if there is an increasing risk of CVEs with multiple COVID-19 infections over that seen in the general population.

Those who had a CVE resulting in death without a family member to report the event would be missed, which would result in an underestimation of the risk of a CVE associated with a COVID-19 illness and/or vaccination. However, if subjects who experienced CVEs or COVID-19 are more likely to complete the survey, this would result in an overestimation of the rate of CVE following COVID-19, increasing the likelihood of finding an association. Lastly, subjects might be more likely to respond to the survey if they have had more healthcare interactions. Thus, mild or asymptomatic cases of COVID-19 that escape being recognized with a positive test result would be underreported, again increasing the chance of finding an association of COVID illness and CVE. Despite these limitations, we found no evidence to discourage COVID-19 vaccination in this population.

While we did not uncover a temporal association between COVID-19 vaccination and CVEs, there was a higher rate of CVEs in those who had been vaccinated. This appears to result from a correlation of age and vaccination. Given the correlation of both aortic diameter with age and of aortic diameter with risk of a CVE in connective tissue disorders [55], the finding that older patients were more likely to have a CVE was not surprising. While we did allow caregivers to enter vaccination data for their children, vaccines for those less than 12 years old were available for less time than they were for adults, and COVID-19 vaccine uptake is correlated with age [56] making this association not unexpected, The association of both vaccination and CVE with age would have increased the chance of discovering an association between the two events, providing further reassurance that if a correlation between vaccination and CVEs exists, it is not large.

Finally, the rate of EAE following COVID-19 vaccination by respondents in our study was similar to (or lower than) the rate reported for the general population [36]. As a concern for serious side effect has been shown to be a significant contributor of vaccine hesitancy [57], this may help vaccine uptake in this population.

## Conclusions

Despite these limitations, our data do not support that there is a substantial increase in self-reported CVEs in the 30 days following COVID-19 vaccination or illness, other than perhaps dysrhythmias following illness, and are in keeping with the recommendation from the Marfan Foundation Professional Advisory Board that all eligible persons be vaccinated for COVID-19.

## Supporting information

S1 Appendix Data dictionary for survey. (PDF)

S1 Fig Cartoon representation. Caption: A web-based survey was made available from November 22, 2021, through March 15, 2022, for those with Marfan Syndrome (MFS), Loeys-Dietz Syndrome (LDS), and vascular Ehlers Danlos Syndrome (vEDS) to enter information regarding their experience with COVID-19 illness, COVID-19 vaccination, and cardiovascular events. 325 respondents (118 with LDS, 176 with MFS, 31 with vEDS) responded to the survey. 254 respondents received a total of 624 vaccination doses, 95 of the respondents reported a COVID-19 illness, and 71 respondents reported a total of 89 cardiovascular events. Using a Cox proportional hazards model with time varying indicators for COVID-19 illness or vaccination, we found no evidence of an increase in CVEs in the 30 days following COVID-19 illness, with the possible exception of dysrhythmia. Following COVID-19 vaccination there was no evidence of an increase in self-reported CVEs in the 30 days post-vaccination.
(PDF)

## Abbreviations

MFS: Marfan Syndrome
LDS: Loeys-Dietz Syndrome
vEDS: vascular Ehlers-Danlos Syndrome
IRB: Institutional Review Board
CVEs: Cardiovascular Events
EAE: Expected Adverse Events
SSPAR: Symptoms that suggest a possible allergic reaction
