# Supplementary material for: Cardiovascular complications in vascular connective tissue disorders after COVID-19 infection and vaccination
Source: PLoS One. 2024 Dec 20;19(12):e0315499. doi: 10.1371/journal.pone.0315499 (PMC11661621; doi:10.1371/journal.pone.0315499)
Supplement: S1 Appendix — (PDF) [file pone.0315499.s001.pdf]

COVID-19 and Connective Tissue Disorders

In order to continue to learn about our community's experience with COVID-19, we are asking for your help with this research study that consists of a brief survey to better understand outcomes.

It is important that we get a balanced view about how people are doing with COVID infection and vaccination. We hope to hear from you if you or your family member have done well since the onset of the pandemic or if you or your family member have had problems. Everyone's experience is valuable.

We continue to recommend the advice given by the CDC and the Marfan Foundation Professional Advisory Board and support vaccine use in the connective tissue disorders community.

To participate in this study on behalf of yourself or for family member, please fill out one study form per individual with a connective tissue disorder. If you are filling out the form for a family member, answer for that family member.

We are asking for participation of:

People living in the United StatesIndividuals older than 18 years with MFS, LDS or VEDSParents of children (ages less than 18 years) with MFS, LDS or VEDSFamily members of individuals with MFS, LDS or VEDS who passed away after Jan 1, 2020If you are filling this out for a child age 5-11, and are intending to vaccinate them for COVID-19, we would ask that you return to this survey about 8 weeks after their second COVID-19 vaccination to fill the survey out. We will send out monthly reminders.

Answering the survey questions implies consent for participation in this study. If you choose to provide your contact information, it implies your consent to contact you for additional information.

Who are you filling this out for?

Date survey is filled out
{[date] text datetime_mdy} ________________________________

Are you filling this out for yourself or a family member?
{[self] radio}

- ◯ {0} family member
- ◯ {1} myself

{[grammar_1] text} ________________________________

{[grammar_2] text} ________________________________

{[grammar_3] text} ________________________________

{[grammar_4] text} ________________________________

{[grammar_5] text} ________________________________

{[grammar_6] text} ________________________________

Some general questions you or your family member.

Have you filled out this survey for [grammar_5] before and are returning to provide updated information?<br><br>{[filled_before] radio}	◯ {0} This is the first time I have filled out this survey for [grammar_6]<br>◯ {1} I have filled out this survey before for [grammar_6], and am returning to provide updated information
What is [grammar_1] date of birth?<br><br>{[dob] text date_mdy}	__________________________________
What is [grammar_1] sex?<br><br>{[sex] radio}	◯ {0} Male<br>◯ {1} Female<br>◯ {2} Prefer not to say
What is [grammar_1] Connective Tissue disorder diagnosis?<br><br>{[dx] radio}	◯ {1} Loeys-Dietz Syndrome (LDS)<br>◯ {2} Marfan Syndrome (MFS)<br>◯ {3} vascular Ehlers-Danlos syndrome (vEDS)<br>◯ {88} other
What would you like to tell us about [grammar_1] diagnosis?<br><br>{[dx_other] textarea}<br>{Branching logic (show if): [dx] = '88'}	____
What is [grammar_1] Loeys-Dietz Syndrome type?<br><br>{[type_lds] radio}<br>{Branching logic (show if): [dx] = '1'}	◯ {1} Type 1 (TGFBR1)<br>◯ {2} Type 2 (TGFBR2)<br>◯ {3} Type 3 (SMAD3)<br>◯ {4} Type 4 (TGFB2)<br>◯ {5} Type 5 (TGFB3)<br>◯ {6} Type 6 (SMAD2)<br>◯ {88} Do not know
[grammar_3] live in the United States?<br><br>{[usa] radio}	◯ {0} No<br>◯ {1} Yes

This survey only includes questions regarding the vaccines and a vaccine schedule available in the United States.

The vaccine [grammar_2] received may not be listed If it occured outside the United States.

{Branching logic (show if): [usa] = '0'}

In this section we are asking about [grammar_1] experience with the COVID-19 Virus

Did [grammar_2] ever have a test for COVID-19 that was positive?

◯ {0} No
◯ {1} Yes

{[covid_pos] radio}

Month Day Year
What was the month, day, and year of [grammar_1] positive COVID-19 test?

Please fill out as much as you remember.

______ ______ ______

{Branching logic (show if): [covid_pos] = '1'}

What were the most severe symptoms [grammar_2] had from the COVID-19 infection?

◯ {0} No symptoms
◯ {1} symptoms similar to a mild cold
◯ {2} symptoms similar to a severe flu but was able to stay home
◯ {3} sick enough to be hospitalized but was on a regular hospital floor
◯ {4} admitted to the intensive care unit
◯ {5} put on a ventilator.
◯ {6} passed away of complications due to COVID

{[covid_symp] radio}
{Branching logic (show if): [covid_pos] = '1'}

Anything else you would like to tell us about [grammar_1] experience with the COVID-19 infection?

____

{[covid_other_info] textarea}
{Branching logic (show if): [covid_pos] = '1'}

In this section will ask about any cardiovascular events that occurred

between January 1, 2019 (a year before the pandemic) and now.

[grammar_4] had a cardiovascular event (listed to the right) or unexplained death between January 1, 2019 (a year before the pandemic) and now?

If so, what was [grammar_1] cardiovascular event?
(choose all that apply)

______ ______

Month Day Year
In what month, day, and year did [grammar_1] cardiovascular event occur?

Please fill out as much as you remember

______ ______ ______

{Branching logic (show if): [cve] = '1'}

You have indicated that the cardiovascular event happened in the same time period as the positive COVID-19 test.

Did the cardiovascular event happen BEFORE or AFTER the positive COVID-19 test?

{[order_cve_covid] radio}
{Branching logic (show if): [covid_pos] = '1' and [cve] = '1' and [covid_pos_year] = [cve_year] and
(
([covid_pos_month] = [cve_month] and
([covid_pos_day] = '99' or [covid_pos_day] = '' or
[cve_day] = '99' or [cve_day] = ''))
or
(([covid_pos_month] = '99' or [covid_pos_month] = ''
or [cve_month] = '99' or [cve_month] = '') and
([covid_pos_day] = '99' or [covid_pos_day] = '' or
[cve_day] = '99' or [cve_day] = ''))
)}

- ◯ {1} The cardiovascular event happened BEFORE the positive COVID test
- ◯ {2} The cardiovascular event happened AFTER the positive COVID test
- ◯ {99} Do not know which happened first

Would you like to provide any more details about [grammar_1] cardiovascular event? Please let us know if you know the following
- name of the blood vessel that caused a problem
- if there was a surgery, was it planned or unplanned?

If [grammar_2] have had more than 1 event, please tell us about it here.
- if you know the date(s) of the additonal event(s), please let us know that as well.

{[cve_other_detail] textarea}
{Branching logic (show if): [cve] = '1'}

This section will ask about [self] experiences with the COVID-19 vaccine.

[grammar_4] received any doses of a COVID-19 vaccine?

{[vac_dose_1] radio}

- ◯ {0} No
- ◯ {1} Yes
- ◯ {99} Do not know

Which vaccine did [grammar_2] receive as the first COVID-19 vaccine dose?

{[vac_type_1] radio}
{Branching logic (show if): [vac_dose_1] = '1'}

- ◯ {1} Moderna
- ◯ {2} Pfizer
- ◯ {3} Johnson and Johnson
- ◯ {99} Do not know

Month Day Year
What month, day, and year did [grammar_2] receive the first COVID-19 vaccine?

Please fill out as much as you remember.

______ ______ ______

{Branching logic (show if): [vac_dose_1] = '1'}

You have indicated that the cardiovascular event happened in the same time period as the first COVID-19 vaccine dose.

Did the cardiovascular event happen BEFORE or AFTER the first COVID-19 vaccine dose?

{[order_cve_vac_1] radio}
{Branching logic (show if): [vac_dose_1] = '1' and [cve] = '1' and [vac_year_1] = [cve_year] and
(
([vac_month_1] = [cve_month] and
([vac_day_1] = '99' or [vac_day_1] = '' or [cve_day] = '99' or [cve_day] = ''))
or
(([vac_month_1] = '99' or [vac_month_1] = '' or [cve_month] = '99' or [cve_month] = '') and
([vac_day_1] = '99' or [vac_day_1] = '' or [cve_day] = '99' or [cve_day] = ''))
)}

- ◯ {1} The cardiovascular event happened BEFORE the first vaccine dose
- ◯ {2} The cardiovascular event happened AFTER the first vaccine dose
- ◯ {99} Do not know which happened first

Did [grammar_2] have symptoms after the first COVID-19 vaccine dose?

{[have_symp_vac_1] radio}
{Branching logic (show if): [vac_dose_1] = '1'}

- ◯ {0} No
- ◯ {1} Yes
- ◯ {99} Do not know

What symptoms did [grammar_2] have after receiving the first COVID-19 vaccine dose?<br>(choose all that apply)<br><br>{[symp_vac_1] checkbox}<br>{Branching logic (show if): [vac_dose_1] = '1' and [have_symp_vac_1] = '1'}	☐ {1} Itching/hives/rash<br>☐ {2} Swelling of face/lips/eyes/throat/tongue<br>☐ {3} Feeling of throat itching/scratchiness/closing/lump in throat<br>☐ {4} Hoarseness of voice<br>☐ {5} runny nose, itchy eyes<br>☐ {6} Coughing spells, wheezing, shortness of breath/difficulty breathing<br>☐ {7} Nausea, vomiting, diarrhea, abdominal cramps<br>☐ {8} Palpitations (heart racing), dizziness<br>☐ {9} passed out/lost consciousness<br>☐ {10} low blood pressure/hypotension<br>☐ {11} Redness/swelling at injection site<br>☐ {12} Fever<br>☐ {13} Chills<br>☐ {14} Fatigue<br>☐ {15} body and/or muscle aches (Myalgia)<br>☐ {16} Joint pains (Arthralgia)<br>☐ {17} Headache<br>☐ {99} Other
What details would you like to provide about [grammar_1] symptoms after the first COVID-19 vaccine dose?<br><br>{[symp_other_vac_1] textarea}<br>{Branching logic (show if): [vac_dose_1] = '1' and [have_symp_vac_1] = '1'}	__________________________________
How soon after receiving the first COVID-19 vaccine did [grammar_2] experience the EARLIEST of these symptoms?<br><br>{[symp_time_vac_1] radio}<br>{Branching logic (show if): [vac_dose_1] = '1' and [have_symp_vac_1] = '1'}	○ {1} Within 1 hour<br>○ {2} Within 6 hours<br>○ {3} Within 24 hours<br>○ {4} After more than 24 hours
Did [grammar_2] need to treat these symptoms?<br><br>{[symp_treat_vac_1] radio}<br>{Branching logic (show if): [vac_dose_1] = '1' and [have_symp_vac_1] = '1'}	○ {0} No<br>○ {1} Yes<br>○ {99} Do not know
What did [grammar_2] use to treat the symptoms?<br>(choose all that apply)<br><br>{[symp_meds_vac_1] checkbox}<br>{Branching logic (show if): [vac_dose_1] = '1' and [have_symp_vac_1] = '1' and [symp_treat_vac_1] = '1'}	☐ {1} Antihistamines (ex: Benadryl, Zyrtec, Claritin, Allegra)<br>☐ {2} Nebulizer or inhaler (ex: Albuterol, Xopenex)<br>☐ {3} Epinephrine (ex: Epipen shot)<br>☐ {4} Steroids<br>☐ {5} Ibuprofen, naproxen (ex: Advil, Aleve)<br>☐ {6} Acetaminophen (ex: Tylenol)<br>☐ {99} Other

What details would you like to provide about [grammar_1] treatment for symptoms after receiving the first COVID-19 vaccine dose?

____

{[symp_meds_other_vac_1] textarea}
{Branching logic (show if): [vac_dose_1] = '1' and [have_symp_vac_1] = '1' and [symp_treat_vac_1] = '1'}

Where were [grammar_1] symptoms treated? (choose all that apply)

{[symp_where_vac_1] checkbox}
{Branching logic (show if): [vac_dose_1] = '1' and [have_symp_vac_1] = '1' and [symp_treat_vac_1] = '1'}

- ☐ {1} Vaccine Administration Center
- ☐ {2} Home
- ☐ {3} Urgent Care
- ☐ {4} Primary care doctor
- ☐ {5} Emergency Department
- ☐ {6} Hospital - regular floor
- ☐ {7} Hospital - Intensive care unit
- ☐ {99} Other

What details would you like to provide about the location for [grammar_2] treatment after receiving the first COVID-19 vaccine dose?

____

{[symp_where_other_vac_1] textarea}
{Branching logic (show if): [vac_dose_1] = '1' and [have_symp_vac_1] = '1' and [symp_treat_vac_1] = '1'}

[grammar_4] received a second dose of the COVID-19 vaccine?

{[vac_dose_2] radio}
{Branching logic (show if): [vac_dose_1] = '1'}

- ◯ {0} No
- ◯ {1} Yes
- ◯ {99} Do not know

Why did [grammar_2] not receive a second COVID-19 vaccine dose?

{[why_no_vac_2] radio}
{Branching logic (show if): [vac_dose_1] = '1' and [vac_dose_2] = '0'}

- ◯ {1} Only one dose is recommended of the vaccine received
- ◯ {2} A second dose is recommended but not received due to scheduling or availability, or not enough time after the first dose has passed yet
- ◯ {3} I had an allergic reaction to the first dose
- ◯ {4} I elected not to receive a second dose
- ◯ {99} Other

Which vaccine did [grammar_2] receive as the second COVID-19 vaccine dose?

{[vac_type_2] radio}
{Branching logic (show if): [vac_dose_1] = '1' and [vac_dose_2] = '1'}

- ◯ {1} Moderna
- ◯ {2} Pfizer
- ◯ {3} Johnson and Johnson
- ◯ {99} Do not know

Month Day Year
What month, day, and year did [grammar_1] receive the second COVID-19 vaccine dose?

Please fill out as much as you remember.

______ ______ ______

{Branching logic (show if): [vac_dose_1] = '1' and [vac_dose_2] = '1'}

You have indicated that the cardiovascular event happened in the same time period as the second COVID-19 vaccine dose.

Did the cardiovascular event happen BEFORE or AFTER the second COVID-19 vaccine dose?

{[order_cve_vac_2] radio}
{Branching logic (show if): [vac_dose_1] = '1' and [vac_dose_2] = '1' and [cve] = '1' and [vac_year_2] = [cve_year] and
(
([vac_month_2] = [cve_month] and
([vac_day_2] = '99' or [vac_day_2] = '' or [cve_day] = '99' or [cve_day] = ''))
or
(([vac_month_2] = '99' or [vac_month_2] = '' or [cve_month] = '99' or [cve_month] = '') and
([vac_day_2] = '99' or [vac_day_2] = '' or [cve_day] = '99' or [cve_day] = ''))
)}

- ◯ {1} The cardiovascular event happened BEFORE the second vaccine dose
- ◯ {2} The cardiovascular event happened AFTER the second vaccine dose
- ◯ {99} Do not know which happened first

Did [grammar_2] have symptoms after the second COVID-19 vaccine dose?

{[have_symp_vac_2] radio}
{Branching logic (show if): [vac_dose_1] = '1' and [vac_dose_2] = '1'}

- ◯ {0} No
- ◯ {1} Yes
- ◯ {99} Do not know

What were [grammar_1] symptoms after receiving the second COVID-19 vaccine dose?
(choose all that apply)

{[symp_vac_2] checkbox}
{Branching logic (show if): [vac_dose_1] = '1' and [vac_dose_2] = '1' and [have_symp_vac_2]='1'}

- ☐ {1} Itching/hives/rash
- ☐ {2} Swelling of face/lips/eyes/throat/tongue
- ☐ {3} Feeling of throat itching/scratchiness/closing/lump in throat
- ☐ {4} Hoarseness of voice
- ☐ {5} Runny nose, itchy eyes
- ☐ {6} Coughing spells, wheezing, shortness of breath/difficulty breathing
- ☐ {7} Nausea, vomiting, diarrhea, abdominal cramps
- ☐ {8} Palpitations (heart racing), dizziness
- ☐ {9} Passed out/lost consciousness
- ☐ {10} Low blood pressure/hypotension
- ☐ {11} Redness/swelling at injection site
- ☐ {12} Fever
- ☐ {13} Chills
- ☐ {14} Fatigue
- ☐ {15} Body and/or muscle aches (Myalgia)
- ☐ {16} Joint pains (Arthralgia)
- ☐ {17} Headache
- ☐ {99} Other

What details would you like to provide about [grammar_1] symptoms after the second COVID-19 vaccine dose?

{[symp_other_vac_2] textarea}
{Branching logic (show if): [vac_dose_1] = '1' and [vac_dose_2] = '1' and [have_symp_vac_2]='1'}

____

How soon after receiving the second COVID-19 vaccine dose did [grammar_2] experience the EARLIEST of these symptoms?<br><br>{[symp_time_vac_2] radio}<br>{Branching logic (show if): [vac_dose_1] = '1' and [vac_dose_2] = '1' and [have_symp_vac_2]='1'}	◯ {1} Within 1 hour<br>◯ {2} Within 6 hours<br>◯ {3} Within 24 hours<br>◯ {4} After more than 24 hours
Did [grammar_2] need to treat these symptoms?<br><br>{[symp_treat_vac_2] radio}<br>{Branching logic (show if): [vac_dose_1] = '1' and [vac_dose_2] = '1' and [have_symp_vac_2]='1'}	◯ {0} No<br>◯ {1} Yes<br>◯ {99} Do not know
What did [grammar_2] use to treat the symptoms? (choose all that apply)<br><br>{[symp_meds_vac_2] checkbox}<br>{Branching logic (show if): [vac_dose_1] = '1' and [vac_dose_2] = '1' and [have_symp_vac_2]='1' and [symp_treat_vac_2] = '1'}	☐ {1} Antihistamines (ex: Benadryl, Zyrtec, Claritin, Allegra)<br>☐ {2} Nebulizer or inhaler (ex: Albuterol, Xopenex)<br>☐ {3} Epinephrine (ex: Epipen shot)<br>☐ {4} Steroids<br>☐ {5} Ibuprofen, naproxen (ex: Advil, Aleve)<br>☐ {6} Acetaminophen (ex: Tylenol)<br>☐ {99} Other
What details would you like to provide about [grammar_1] treatment for symptoms after receiving the second COVID-19 vaccine dose?<br><br>{[symp_meds_other_vac_2] textarea}<br>{Branching logic (show if): [vac_dose_1] = '1' and [vac_dose_2] = '1' and [have_symp_vac_2]='1' and [symp_treat_vac_2] = '1'}	________________________________
Where were [grammar_1] symptoms treated? (choose all that apply)<br><br>{[symp_where_vac_2] checkbox}<br>{Branching logic (show if): [vac_dose_1] = '1' and [vac_dose_2] = '1' and [have_symp_vac_2]='1' and [symp_treat_vac_2] = '1'}	☐ {1} Vaccine Administration Center<br>☐ {2} Home<br>☐ {3} Urgent Care<br>☐ {4} Primary care doctor<br>☐ {5} Emergency Department<br>☐ {6} Hospital - regular floor<br>☐ {7} Hospital - Intensive care unit<br>☐ {99} Other
What details would you like to provide about the location for [grammar_2] treatment after receiving the second COVID-19 vaccine dose?<br><br>{[symp_where_other_vac_2] textarea}<br>{Branching logic (show if): [vac_dose_1] = '1' and [vac_dose_2] = '1' and [have_symp_vac_2]='1' and [symp_treat_vac_2] = '1'}	________________________________

[grammar_4] received a third dose of the COVID-19 vaccine (booster)?<br><br>{[vac_dose_3] radio}<br>{Branching logic (show if): [vac_dose_1] = '1' and [vac_dose_2] = '1'}	◯ {0} No<br>◯ {1} Yes<br>◯ {99} Do not know
Why did [grammar_2] not receive a third COVID-19 vaccine dose (booster)?<br><br>{[why_no_vac_3] radio}<br>{Branching logic (show if): [vac_dose_1] = '1' and [vac_dose_2] = '1' and [vac_dose_3] = '0'}	◯ {1} A third dose has not been recommended<br>◯ {2} A third dose is recommended but not received due to scheduling or availability, or not enough time after the second dose has passed yet<br>◯ {3} I had an allergic reaction to a previous dose<br>◯ {4} I elected not to receive a third dose<br>◯ {99} Other
Which vaccine did [grammar_2] receive as the third COVID-19 vaccine dose?<br><br>{[vac_type_3] radio}<br>{Branching logic (show if): [vac_dose_1] = '1' and [vac_dose_2] = '1' and [vac_dose_3] = '1'}	◯ {1} Moderna<br>◯ {2} Pfizer<br>◯ {3} Johnson and Johnson<br>◯ {99} Do not know

Month Day Year
What month, day, and year did [grammar_2] receive your third COVID-19 vaccine dose?

Please fill out as much as you remember.

______ ______ ______

{Branching logic (show if): [vac_dose_1] = '1' and [vac_dose_2] = '1' and [vac_dose_3] = '1'}

You have indicated that the cardiovascular event happened in the same time period as the third COVID-19 vaccine dose.<br><br>Did the cardiovascular event happen BEFORE or AFTER the third COVID-19 vaccine dose?<br><br>{[order_cve_vac_3] radio}<br>{Branching logic (show if): [vac_dose_1]= '1' and [vac_dose_2] = '1' and [vac_dose_3] = '1' and [cve] = '1' and [vac_year_3] = [cve_year] and<br>(<br>([vac_month_3] = [cve_month] and ([vac_day_3] = '99' or [vac_day_3] = '' or [cve_day] = '99' or [cve_day] = ''))<br>or<br>(([vac_month_3] = '99' or [vac_month_3] = '' or [cve_month] = '99' or [cve_month] = '') and ([vac_day_3] = '99' or [vac_day_3] = '' or [cve_day] = '99' or [cve_day] = ''))<br>)}	◯ {1} The cardiovascular event happened BEFORE the third vaccine dose<br>◯ {2} The cardiovascular event happened AFTER the third vaccine dose<br>◯ {99} Do not know which happened first

Did [grammar_2] have symptoms after the third COVID-19 vaccine dose?

{[have_symp_vac_3] radio}
{Branching logic (show if): [vac_dose_1] = '1' and [vac_dose_2] = '1' and [vac_dose_3] = '1'}

- {0} No
- {1} Yes
- {99} Do not know

What were [grammar_1] symptoms after receiving the third COVID-19 vaccine dose?
(choose all that apply)

{[symp_vac_3] checkbox}
{Branching logic (show if): [vac_dose_1] = '1' and [vac_dose_2] = '1' and [vac_dose_3] = '1' and [have_symp_vac_3] = '1'}

- {1} Itching/hives/rash
- {2} Swelling of face/lips/eyes/throat/tongue
- {3} Feeling of throat itching/scratchiness/closing/lump in throat
- {4} Hoarseness of voice
- {5} runny nose, itchy eyes
- {6} Coughing spells, wheezing, shortness of breath/difficulty breathing
- {7} Nausea, vomiting, diarrhea, abdominal cramps
- {8} Palpitations (heart racing), dizziness
- {9} passed out/lost consciousness
- {10} low blood pressure/hypotension
- {11} Redness/swelling at injection site
- {12} Fever
- {13} Chills
- {14} Fatigue
- {15} body and/or muscle aches (Myalgia)
- {16} Joint pains (Arthralgia)
- {17} Headache
- {99} Other

What details would you like to provide about [grammar_1] symptoms after the third COVID-19 vaccine dose?

{[symp_other_vac_3] textarea}
{Branching logic (show if): [vac_dose_1] = '1' and [vac_dose_2] = '1' and [vac_dose_3] = '1' and [have_symp_vac_3] = '1'}

____

How soon after receiving the third COVID-19 vaccine dose did [grammar_2] experience the EARLIEST of these symptoms?

{[symp_time_vac_3] radio}
{Branching logic (show if): [vac_dose_1] = '1' and [vac_dose_2] = '1' and [vac_dose_3] = '1' and [have_symp_vac_3] = '1'}

- {1} Within 1 hour
- {2} Within 6 hours
- {3} Within 24 hours
- {4} After more than 24 hours

Did [grammar_2] need to treat these symptoms?

{[symp_treat_vac_3] radio}
{Branching logic (show if): [vac_dose_1] = '1' and [vac_dose_2] = '1' and [vac_dose_3] = '1' and [have_symp_vac_3] = '1'}

- {0} No
- {1} Yes
- {99} Do not know

What did [grammar_2] use to treat the symptoms? (choose all that apply)<br><br>{[symp_meds_vac_3] checkbox}<br>{Branching logic (show if): [vac_dose_1] = '1' and [vac_dose_2] = '1' and [vac_dose_3] = '1' and [have_symp_vac_3] = '1' and [symp_treat_vac_3] = '1'}	☐ {1} Antihistamines (ex: Benadryl, Zyrtec, Claritin, Allegra)<br>☐ {2} Nebulizer or inhaler (ex: Albuterol, Xopenex)<br>☐ {3} Epinephrine (ex: Epipen shot)<br>☐ {4} Steroids<br>☐ {5} Ibuprofen, naproxen (ex: Advil, Aleve)<br>☐ {6} Acetaminophen (ex: Tylenol)<br>☐ {99} Other
What details would you like to provide about [grammar_1] treatment for symptoms after receiving the third COVID-19 vaccine?<br><br>{[symp_meds_other_vac_3] textarea}<br>{Branching logic (show if): [vac_dose_1] = '1' and [vac_dose_2] = '1' and [vac_dose_3] = '1' and [have_symp_vac_3] = '1' and [symp_treat_vac_3] = '1'}	______________________________
Where were [grammar_1] symptoms treated? (choose all that apply)<br><br>{[symp_where_vac_3] checkbox}<br>{Branching logic (show if): [vac_dose_1] = '1' and [vac_dose_2] = '1' and [vac_dose_3] = '1' and [have_symp_vac_3] = '1' and [symp_treat_vac_3] = '1'}	☐ {1} Vaccine Administration Center<br>☐ {2} Home<br>☐ {3} Urgent Care<br>☐ {4} Primary care doctor<br>☐ {5} Emergency Department<br>☐ {6} Hospital - regular floor<br>☐ {7} Hospital - Intensive care unit<br>☐ {99} Other
What details would you like to provide about the location for [grammar_2] treatment after receiving the third COVID-19 vaccine dose?<br><br>{[symp_where_other_vac_3] textarea}<br>{Branching logic (show if): [vac_dose_1] = '1' and [vac_dose_2] = '1' and [vac_dose_3] = '1' and [have_symp_vac_3] = '1' and [symp_treat_vac_3] = '1'}	______________________________

Thank you!

If we can contact you for more information please enter your phone and/or email below

Phone number

____

{[phone] text phone}

email

____

{[email] text email}

Open study to measure COVID-19 antibodies in LDS

The NIH has an open study examining COVID antibodies in patients with certain genetic disorders including Loeys-Dietz syndrome. This study entails you having a finger-stick kit mailed to you. You would collect a drop of blood (similar to the finger prick that a diabetic does to check their blood sugar) and mail the pre-paid kit back to the NIH. Results from the testing are not given back to participants. If you would like more information, please contact Dr. Emily Ricotta at the NIH (emily.ricotta@nih.gov).

{Branching logic (show if): [dx] = '1'}
