# Supplementary figures and images for: Cardiovascular complications in vascular connective tissue disorders after COVID-19 infection and vaccination
Source: PLoS One. 2024 Dec 20;19(12):e0315499. doi: 10.1371/journal.pone.0315499 (PMC11661621; doi:10.1371/journal.pone.0315499)

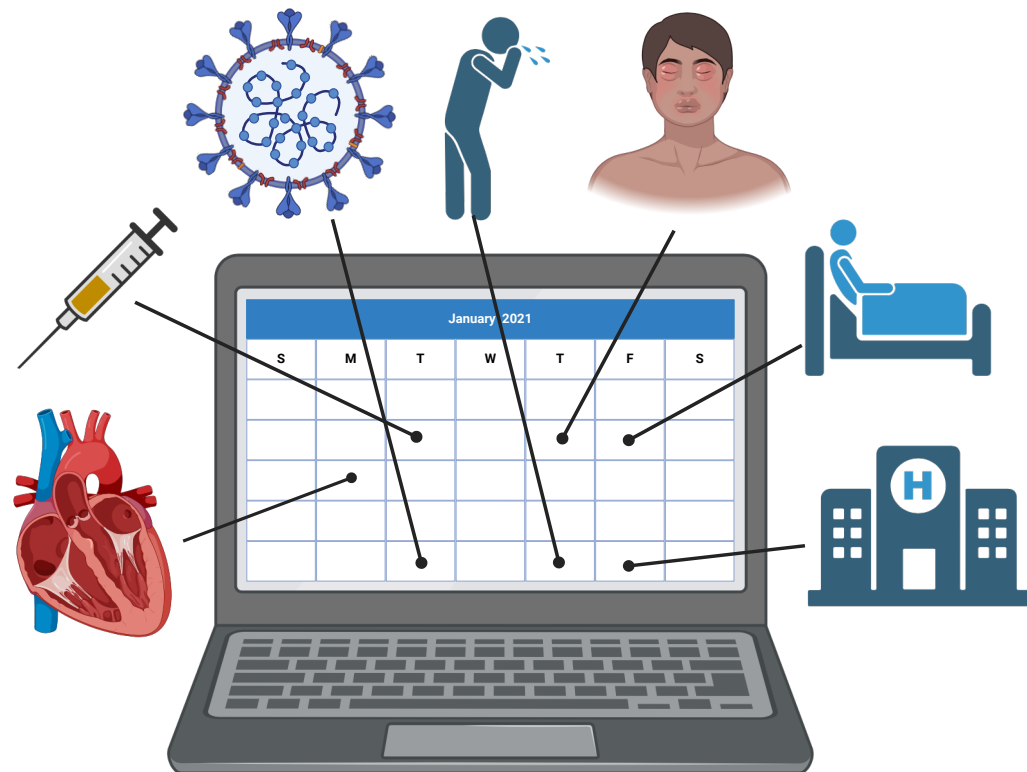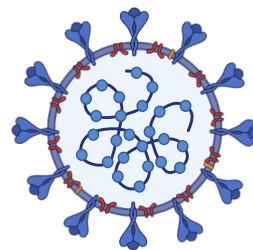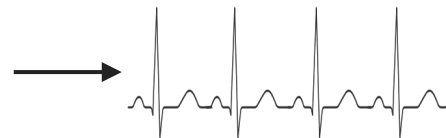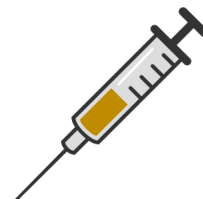

No  
Associations

Supplement: S1 Fig — Caption: A web-based survey was made available from November 22, 2021, through March 15, 2022, for those with Marfan Syndrome (MFS), Loeys-Dietz Syndrome (LDS), and vascular Ehlers Danlos Syndrome (vEDS) to enter information regarding their experience with COVID-19 illness, COVID-19 vaccination, and cardiovascular events. 325 respondents (118 with LDS, 176 with MFS, 31 with vEDS) responded to the survey. 254 respondents received a total of 624 vaccination doses, 95 of the respondents reported a COVID-19 illness, and 71 respondents reported a total of 89 cardiovascular events. Using a Cox proportional hazards model with time varying indicators for COVID-19 illness or vaccination, we found no evidence of an increase in CVEs in the 30 days following COVID-19 illness, with the possible exception of dysrhythmia. Following COVID-19 vaccination there was no evidence of an increase in self-reported CVEs in the 30 days post-vaccination. (PDF) [file pone.0315499.s002.pdf]
